# Transmission saisonnière du paludisme au niveau de la vallée du fleuve Sénégal: cas de la ville de Kaédi-Mauritanie

**DOI:** 10.11604/pamj.2019.34.185.20011

**Published:** 2019-12-06

**Authors:** Ousmane Ba, Aïchetou Sow, Hampâté Ba, Sid’Ahmed Dahdi, Baidy Lo

**Affiliations:** 1 Faculté de Médecine, Université de Nouakchott Al Aasriya, Nouakchott, Mauritanie; 2 Institut National de Recherches en Santé Publique (INRSP), Nouakchott, Mauritanie

**Keywords:** Paludisme, *Plasmodium falciparum*, *Plasmodium malariae*, anopheles, saison pluvieuse, saison sèche, Kaédi, Mauritanie, Malaria, Plasmodium falciparum, Plasmodium malariae, anopheles, rainy season, dry season, Kaedi, Mauritania

## Abstract

**Introduction:**

Face aux changements environnementaux et climatiques en cours et prévus, l'épidémiologie du paludisme dans la ville de Kaédi (Mauritanie), riveraine de la vallée du fleuve Sénégal, exige une attention particulière. Des cas de paludisme sont enregistrés dans les établissements de santé tout au long de l'année, avec en moyenne 150 000 cas annuels présomptifs et les conditions climatiques et écologiques actuelles sont favorables à une transmission saisonnière.

**Méthodes:**

Nous avons mené deux enquêtes transversales descriptives dans la ville de Kaédi en septembre 2014 (saison humide) et mai 2015 (saison sèche). Un échantillonnage en grappes a permis de toucher 700 ménages. Tous les membres des ménages ont fait objet d'examen microscopique. Par ailleurs des prospections larvaires, des pulvérisations de la faune matinale et des pauses de pièges nocturnes ont été réalisées.

**Résultats:**

Sur l'ensemble des deux saisons 9313 gouttes épaisses ont été confectionnées, 15 étaient positives soit un indice plasmodique moyen de 0,16%. Parmi eux 12 étaient positives en saison sèche et 3 en saison pluvieuse. L'indice plasmodique moyen a été ainsi de 0,26% et de 0,06% respectivement en saison sèche (n = 4642) et pluvieuse (n = 4671). En saison pluvieuse, les prévalences ont été de 0,04% (2/4671) et 0,02% (1/4671) respectivement pour *Plasmodium malariae* et *Plasmodium falciparum*. En saison sèche le *Plasmodium falciparum* est la seule espèce rencontrée. Les investigations entomologiques ont montré la présence d'une seule espèce d'anophèle, il s'agit d'*Anopheles gambiae*, dont deux (2) en saison pluvieuse et six (6) en saison sèche. Les prospections des gites larvaires ont montré que la faune larvaire est dominée par les larves du genre Culex (99,6%). La faune anophelienne (0,4%) a été récoltée uniquement pendant la saison sèche.

**Conclusion:**

Malgré la faible transmission du paludisme dans la ville de Kaédi, dans un contexte d'absence de pluviométrie, les autorités sanitaires doivent entrevoir une stratégie de pré-élimination du paludisme dans les wilayas riveraines du fleuve Sénégal.

## Abstract (en)

**Introduction:**

In the face of environmental and climatic changes both ongoing and planned, the epidemiology of malaria in the city of Kaedi (Mauritania), along the Senegal River Valley, requires special attention. Some cases of malaria have been registered in the health facilities throughout the year, with an average of 150,000 annual suspected cases and climatic and ecological conditions which are now favorable for seasonal transmission.

**Methods:**

We conducted two cross-sectional descriptive surveys in the city of Kaedi in September 2014 (wet season) and in May 2015 (dry season). Our cluster sampling involved 700 households. Microscopic examination was performed in all household members. Furthermore, larval surveys, early morning wildlife spraying and nocturnal traps breaking were performed.

**Results:**

During both seasons, 9.313 thick smears were manufactured, 15 were positive, with a plasmodium prevalence rate of 0.16%. Among these, 12 were positive in the dry season and 3 in the rainy season. Plasmodium prevalence rate was 0.26% and 0.06% respectively in the dry season (n = 4642) and in the wet season (n = 4671). In the rainy season, rates were 0.04% (2/4671) and 0.02% (1/4671) respectively for Plasmodium malariae and Plasmodium falciparum. The only species found in the dry season was Plasmodium falciparum. Entomological investigations showed the presence of a single species of Anopheles mosquito, Anopheles gambia (two in the rainy season and six in the dry season). Larval surveys showed that the larval fauna was dominated by Culex larvae (99.6%). Anopheles larvae (0.4%) were collected only during the dry season.

**Conclusion:**

Despite low malaria transmission in the city of Kaedi, in a context of lack of rainfall, health authorities should implement a strategy for malaria elimination in the wilayas of the Senegal River.

## Introduction

Le paludisme est l'une des plus fréquentes maladies infectieuses. Les infections à *Plasmodium falciparum* peuvent être mortelles et les enfants africains de moins de 5 ans en sont souvent les victimes [1]. En Afrique et en Asie du sud-Est, cette maladie demeure un problème majeur de santé publique. En Mauritanie, le paludisme occupe le troisième rang des motifs de consultation au niveau national, après les infections respiratoires et les maladies diarrhéiques [2]. Une incidence moyenne annuelle de 150 000 cas présomptifs, est enregistrée dans les structures sanitaires des zones de transmission, selon le Programme National de Lutte contre le Paludisme (PNLP). Quatre des cinq espèces plasmodiales responsables du paludisme humain (*P. falciparum, P. malariae, P. vivax et P. ovale*) y sont rencontrées [3-5]. Le *Plasmodium falciparum* est l'espèce plasmodiale dominante au cours de la saison des pluies, en particulier dans la partie Sud où se situe la ville de Kaédi. Toutefois, une forte prédominance du *Plasmodium vivax* est observée à Nouakchott et dans les Wilayas du Nord [6-8]. La faune anophélienne décroit du Sud au Nord du pays et d'une année à l'autre [9]. L'espèce anophélienne prédominante est *Anopheles Gambiæ s.l* représentée par *An. arabiensis* [10]. Face aux changements environnementaux et climatiques en cours et prévus, l'épidémiologie du paludisme dans la ville de Kaédi, riveraine de la vallée du fleuve Sénégal, exige une attention particulière, notamment en saison humide où des inondations sont enregistrées. Devant aussi la rareté des données épidémiologiques, cette étude a été conçue pour évaluer la situation réelle de la transmission, ce qui permettra aux autorités sanitaires d'optimiser les stratégies de la lutte antipaludique. L'objectif de cette étude est de décrire la transmission du paludisme dans la ville de Kaédi (Mauritanie), au cours de la saison humide de l'année 2014 et saison sèche 2015.

## Méthodes

**Figure 1:**
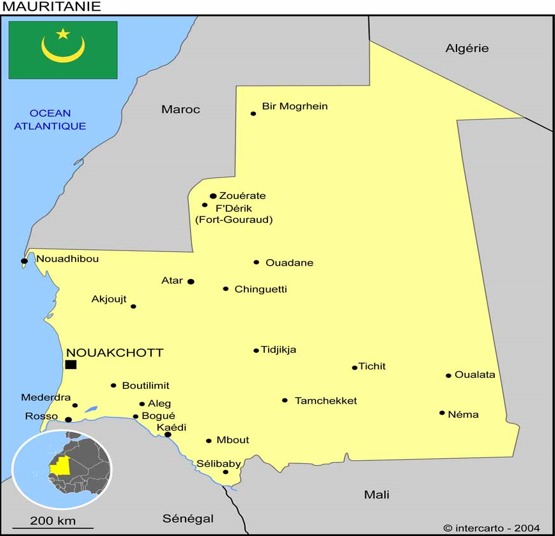
Localisation de Kaédi sur la carte de la République Islamique de Mauritanie

**Cadre d'étude:** Kaédi est une ville du sud de la Mauritanie capitale de la willaya du Gorgol, distante d'environ 430 km de la capitale Nouakchott. Elle est située sur la rive droite du fleuve Sénégal (Figure 1). Le lit du fleuve et les cuvettes de décantation à texture sablo-limoneuse où se déversent périodiquement les crues, sont recouverts d'une épaisse couche argileuse cultivée en riz ou en mil. La ville de Kaédi est située dans une région traditionnellement agricole, ce qui explique en particulier la permanence d'un système agro-pastoral productif. Le climat se caractérise par une opposition saisonnière (une longue saison sèche et une courte saison de pluies: actuellement entre 50 et 70 mm/an) due aux conditions atmosphériques qui règnent sur toute l'Afrique Occidentale. Entre décembre et janvier, les températures peuvent descendre jusqu'à 16°C. Dès le mois de février, elles s'élèvent pour atteindre le maximum en mai (plus de 40°C).

**Collecte des données démographiques et parasitologiques, et cliniques:** nous avons effectué une étude transversale descriptive qui s'est déroulée en deux saisons sur une période de 1 mois: l'une en saison humide ou pluvieuse, septembre 2014 durant 15 jours et l'autre en saison sèche, mai 2015 durant 12 jours. Cette étude fait partie d'une enquête plus large de ménages dont le calcul de la taille de l'échantillon (n) a été basé sur les données suivantes:

n = y^2^*p (1-p)*c/i^2^

avec p = prévalence attendue pour la maladie étudiée (p = 0.50%), α = risque, généralement égale à 5% (écart type γ = 1,96), i = défaut de précision (i = 5%), et c = coefficient de correction (c = 2); soit environ 770 ménages.

**Figure 2:**
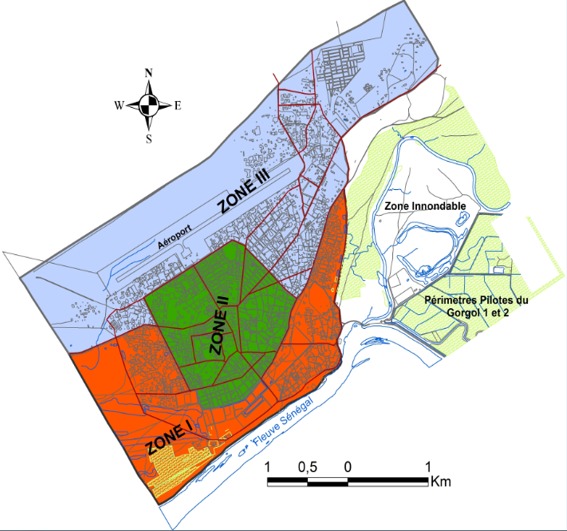
Localisation des trois zones du risque d’inondation sur la carte spatiale de la ville de Kaédi

Le choix de la prévalence de 50% est motivé par le fait qu'il s'agissait d'une étude pilote sans précédente où nous voulions maximaliser l'effectif. Et donc le recours à l'unité “ménage”, dans une étude portant sur le paludisme qui est un problème de santé d'individus et non des ménages, est motivé par les autres composantes (volets socioéconomique et comportementale) de cette grande étude ménage. Pour le choix des ménages, un échantillonnage en grappes a été réalisé et la ville a été subdivisée en dix sous unités spatiales à l'aide d'une carte de la ville et avec la contribution des populations locales. Chaque équipe d'enquêteurs a travaillé dans l'une des dix sous-unités, en visitant soixante-dix-sept ménages, identifiés de façon aléatoire, selon la technique du programme élargi de vaccination (PEV) de l'OMS. Ainsi, on a pris un point de départ bien connu dans la sous-unité (ou sous quartier). La première concession a été choisie en faisant tourner une bouteille autour d'elle-même. Puis on a parcouru en spirale, les concessions du sous-quartier dans le sens des aiguilles d'une montre, en choisissant une entrée (porte) sur quatre, en alternant chaque fois du côté droit au côté gauche de la rue, si la possibilité existe (autrement, on est resté sur le même côté). La concession peut être constituée d'un ou plusieurs ménages. Un ménage sur quatre a été choisi de façon aléatoire dans chaque concession, en attribuant aux ménages d'une concession des numéros et faisant un tirage sans remise. Par ailleurs pour les besoins de notre étude, la ville de Kaédi, comportant 11 quartiers, a été divisée en trois zones: zone I, zone II, zone III, du plus inondable au moins inondable (Figure 2). L'enquête a ciblé systématiquement tous les membres des ménages, et a concerné les participants exprimant leur accord par la signature du formulaire de consentement éclairé. Ainsi l'équipe médicale a visité tous les ménages sélectionnés afin de remplir des fiches d'enquête; de prendre la température; de confectionner des gouttes épaisses et des frottis. Deux gouttes de sang capillaire ont été recueillies sur le bout du doigt de chaque participant. Elles ont permis de confectionner une goutte épaisse et un frottis mince. La coloration GIEMSA diluée à 10%, préconisée par l'OMS, a été utilisée pour colorer les lames au Laboratoire de Parasitologie-Mycologie de l'Institut National de Recherches en Santé Publique (INRSP). Les lectures au microscope à l'huile à immersion (x 100) ont permis la mise en évidence des gouttes épaisses positives, l'identification des espèces plasmodiales responsables de l'infection palustre et la détermination de la densité parasitaire. Pour calculer la densité parasitaire (exprimée en nombre de parasites asexués par μl de sang), le nombre de parasites asexués comptés (par rapport à 200 ou 500 globules blancs) a été divisé par le nombre de leucocytes comptés et multiplié par un supposé nombre de globules blancs de 8000 par μl. Par ailleurs un contrôle de qualité de 10% des lames a été effectué par un microscopiste sénior.

**Figure 3:**
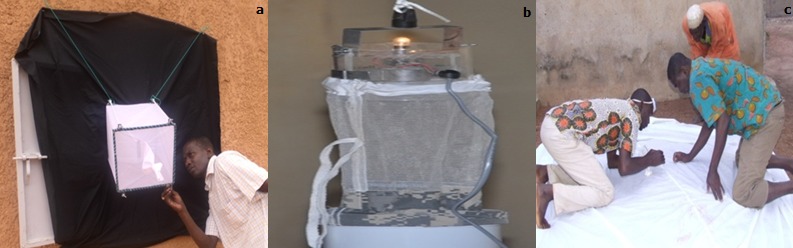
Collecte des imagos au niveau des ménages de Kaédi en 2014: a) pièges fenêtres; b) pièges lumineux; c) faune matinale

**Collecte de données entomologiques:** deux techniques d'échantillonnage de moustiques adultes ont été utilisées: la capture aux pièges (pièges fenêtres (PF) et pièges lumineux (PL)) et la collecte de la faune au repos dans les habitations humaines par pulvérisation intra-domiciliaire (PID) (Figure 3). Nous avons choisi 12 maisons reparties dans les trois zones de la ville au niveau desquels nous avons posé 12 pièges, chaque piège a passé 3 nuits. Des pièges de type CDC (Center of Disease Control, Atlanta) associés avec un dormeur sous moustiquaire ont été utilisés, ces pièges ont fonctionné de 19 heures à 6 heures du matin. Pour la capture de la faune matinale, vingt-et-une (21) maisons (y compris les hangars) ont été choisies pour la collecte des moustiques. Cette méthode d'échantillonnage consiste à collecter les moustiques dans leurs lieux de repos, tôt le matin avant l'ouverture des portes et fenêtres des chambres et salons sélectionnés la veille. La technique utilisée est celle des récoltes après pulvérisation de pyrèthre. Après avoir recouvert le plancher de draps blancs et fermé toutes les issues de la pièce, on procède à une pulvérisation de pyréthrinoïdes (YOTOX^®^). Au bout de 10 minutes d'attente, les moustiques tombés sont prélevés à l'aide de pinces souples dans des boîtes de pétri. Le nombre de chambre à pulvériser a été choisi en fonction de la taille du quartier. On a choisi de 3 à 11 chambres pulvérisé par quartier. Par ailleurs, la méthode du “dipping” a été utilisée pour la récolte des larves et nymphes de moustiques. Elle a consisté à donner 25 coups de bac (0,75 litre d'eau). Les larves et nymphes récoltées ont été dénombrées en fonction du genre (Anopheles, Culex et Aedes) et des stades. Les larves et les imagos ont été identifiés sur la base des caractères morphologiques.

**Analyse des données:** la saisie et l'analyse des données ont été faites à partir du logiciel Epi DATA et du logiciel SPSS. Les proportions ont été comparées à l'aide du test du chi carré et le niveau de signification statistique a été fixé à p < 0,05.

**Considérations éthiques:** la présente étude a été examinée et approuvée par le comité éthique du Ministère Mauritanien de la Santé. Le but de l'étude a été expliqué en dialecte local aux chefs des ménages sélectionnés, qui ont signé les différents consentements éclairés. Par ailleurs tous les enfants avec un frottis positif et/ou un résultat de TDR, quel que soit l'espèce plasmodiale, ont été immédiatement référés au plus proche centre de santé pour traitement antipaludique.

## Résultats

**Données parasitologiques:** au total, 9313 individus ont été inclus dans notre étude et le sex-ratio était de 0,64. Sur l'ensemble des 9313 gouttes épaisses confectionnées, 15 étaient positives soit un indice plasmodique moyen de 0,16%, 12 étaient positives en saison sèche et 3 en saison pluvieuse. L'indice plasmodique moyen a été ainsi de 0,26% et de 0,06% respectivement en saison sèche (n = 4642) et pluvieuse (n = 4671). Mais quel que soit la saison, l'indice plasmodique a varié de façon significative (p = 0,019). Chez les sujets de sexe féminin, l'indice plasmodique moyen a été de 0,07% (2/2698) en saison pluvieuse et 0.45% (12/2673) en saison sèche. Chez les sujets de sexe masculin l'indice plasmodique moyen a été de 0,05% (1/1801) et de 0.12% (2/1680) respectivement en saison pluvieuse et sèche, l'indice plasmodique n'a pas varié de façon significative selon le sexe (p = 0,16). Le Tableau 1 montre la répartition de l'indice plasmodiale par tranche d'âge selon les saisons avec un cas de goutte épaisse positive (GE+) en saison sèche dont l'âge est inconnu. L'indice plasmodique n'a pas significativement varié en fonction de l'âge (P = 0,51). Deux espèces plasmodiales ont été identifiées en saison pluvieuse, les valeurs ont été de (2/3) et (1/3) respectivement pour *Plasmodium malariae* et *Plasmodium falciparum*. En revanche *Plasmodium falciparum* est la seule espèce plasmodiale rencontrée en saison sèche. La répartition par classe d'âge des densités parasitaires a montré que les charges parasitaires les plus élevées (500-4999 parasites par μl de sang) ont été principalement observées dans les classes d'âge 0-5 et > 18 ans. En revanche aucune classe d'âge n'a enregistré une charge parasitaire < 50 parasites par μl de sang. L'essentiel des patients ayant une goutte épaisse positive (10/15) ont une charge parasitaire entre 50 et 499 parasites par μl de sang. Le Tableau 2 montre l'évolution des températures corporelles et des gouttes épaisses positives selon les saisons pendant notre étude. Les variations de température étaient significative durant la même saison (p = 0,000). Sur les onze (11) quartiers sélectionnés durant l'enquête un total de 15 gouttes épaisses positives a été noté. Parmi elles, 12 en saison sèche avec une répartition inégale au niveau des différents quartiers. Kebe était le quartier où le taux était le plus élevé avec 4 gouttes épaisses positives, tandis qu'en saison pluvieuse le quartier de Tantadji avait la fréquence la plus élevée.

**Tableau 1:** Répartition de l’indice plasmodiale par tranche d’âge selon les saisons

GE+ saisons	Tranches d’âge				P-value
	0-5 ans	5-10 ans	10-18 ans	> 18 ans	
**GE+ en saison pluvieuse**	0.2% (1/508)	0.1% (1/945)	0%	0.06% (1/1783)	0.51
**GE+ en saison sèche**	0.34% (3/873)	0.4% (3/797)	0.1% (1/887)	0.2% (4/1797)	0.51

**Tableau 2:** Évolution des températures corporelles et des gouttes épaisses positives selon les saisons à Kaédi

	Tranches de température					P-value
	˂37.5°C	37.5-38°C	38-39°C	> 39°C	NI	
**Pluvieuse**	4174 (89.4%)	247 (5.3%)	76 (1.6%)	15 (0.3%)	159 (3.4%)	p<10^-3^
**Pluvieuse/GE+**	2	0	1	0	0	p>0,05
**Sèche**	3999 (86.1%)	119 (2.6%)	53 (1.1%)	14 (0.3%)	457 (9.8%)	
**Sèche/GE+**	6	0	3	3	0	

**NI =** Individus dont les températures sont inconnues

**Données entomologiques:** cinquante-trois (53) gîtes larvaires (41 naturels et 12 anthropiques) ont été prospectés lors de l'investigation dont vingt-six (26) lors de la saison pluvieuse et vingt-sept (27) en saison sèche. La faune larvaire était dominée par les larves du genre Culex (99,6%). La faune anophelienne (0,4%) a été récoltée uniquement pendant la saison sèche. La faune larvaire était plus abondante lors de la saison sèche (99%) que pendant la saison pluvieuse (1%). Huit (8) moustiques *Anopheles gambiae* (7 femelles contre 1 mâle) ont été récoltés au cours de l'étude, dont deux (2) en saison pluvieuse et six (6) en saison sèche. En revanche 103 moustiques du genre Culex ont été récoltés, dont 16 en saison pluvieuse et 87 en saison sèche (Tableau 3).

**Tableau 3:** Composition de la faune culicidienne selon la méthode de collecte et la saison à Kaédi

	Méthodes de collectes				
	Saison pluvieuse		Saison sèche		
Espèces	PF	PID	PF	PID	PL
***An. gambiae***	0	2	1	2	3
***Culex***	4	12	0	14	73
**Total**	4	14	1	16	76

## Discussion

En Mauritanie, le paludisme sévit sous forme d'épidémie, la transmission est dans l'ensemble faible [10]. En ce qui concerne notre étude, la prévalence globale a été de 0,16%, elle a été plus faible en saison pluvieuse. Cette faiblesse de la prévalence en saison pluvieuse, par rapport à la saison sèche, était liée à l'absence de la pluviométrie au moment de l'investigation (arrivée tardive des pluies par rapport à la saison). Cependant quel que soit la saison, la prévalence du paludisme est globalement faible, ce qui est conforme à la situation décrite dans les études de faciès épidémiologiques [10]. Le paludisme est instable en Mauritanie et la stratification des zones de transmission palustres a été déterminée en fonction des résultats obtenus à travers des enquêtes entomologiques, parasitologique et des contextes écologiques ayant des implications sur la morbidité et la mortalité palustres. Ainsi, trois zones (ou strates) de faciès ont été identifiées. La zone sahélienne où se localise la ville de Kaédi, caractérisée par une transmission saisonnière faible avec un arrêt de transmission durant plusieurs années [5, 11]. Dans cette zone, des études en 2011 et 2013 avaient trouvé des prévalences de 1,71% et 2,37% respectivement en saison sèche et pluvieuse.

Ainsi à l'instar de notre investigation, ces études de faciès épidémiologiques ont montré que la transmission du paludisme reste généralement faible en Mauritanie et particulièrement à Kaédi. Il faut noter que cette étude de faciès épidémiologique a concerné les patients fébriles et non fébriles [10]. Touray S *et al.* ont rapporté une prévalence nulle (0,00%) chez 371 enfants en saison sèche dans la ville de Kaédi [12]. Cependant, Cortes *et al.* avaient trouvé une prévalence de 25,49% chez les présumés paludéens au niveau des structures sanitaires de Kaédi [13]. Mais la ville était plus humide et l'étude avait ciblé les consultants fébriles. D'autres auteurs ont rapporté une faible transmission du paludisme au niveau des trois zones de faciès épidémiologiques, donc au niveau de la vallée du fleuve Sénégal [14, 15]. Globalement, l'indice plasmodique n'a pas varié de façon significative selon le sexe (p = 0,16). Cependant, il a été plus élevé chez les sujets de sexe féminin en saison sèche. L'indice plasmodique n'a pas significativement varié en fonction de l'âge (p = 0,51), ce qui traduit la lenteur de l'acquisition ou l'absence de l'immunité antipalustre stable en zone de faible transmission. Deux espèces plasmodiales, *P. malariae* et *P. falciparum* ont été identifiées avec un taux faible qui ne permet pas d'apprécier leur fréquence. De même, des études antérieures avaient mis en évidence la présence de ces deux espèces [6, 15], bien que d'autres avaient souligné la prédominance du *Plasmodium falciparum* au sud du pays [12, 15]. Les espèces plasmodiales rencontrées en saison pluvieuse, alors que la pluie n'était pas encore au rendez-vous, en effet il y'avait un retard de pluviométrie, peuvent s'expliquer par des infections anciennes (des reviviscences très tardives du *Plasmodium malariae*) ou la persistance d'une faible transmission au niveau des quartiers riverains du fleuve Sénégal ou des cours d'eau. Il existe des fièvres non palustres, ce qui laisse présager l'existence d'étiologies non palustres. Près de 90% de notre échantillon étaient apyrétique, cette absence massive de fièvre explique les taux faibles de gouttes épaisses positives. Aucune classe d'âge n'a enregistré une charge parasitaire < 50 parasites par μl de sang. Mais l'évolution des charges parasitaires a montré une fluctuation en fonction de l'âge. Les charges parasitaires élevées (500-4999 parasites par μl de sang) ont été notées seulement dans les classes d'âge 0-5 ans et > 18 ans. Ce qui traduit dans une certaine mesure l'absence de prémunition car en zone de faible transmission il est difficile d'acquérir une immunité protectrice stable. L'instabilité de la transmission du paludisme en Mauritanie favorise le mode épidémique.

Ouldahmedousalem *et al.* ont trouvé de fortes densités parasitaires dans tous les groupes d'âges en 2010 en Hodh el Gharbi, wilaya Est du pays [16] où la transmission du paludisme est faible, ce qui confirme l'absence de prémunition. D'autres auteurs ont rapporté des charges parasitaires élevées chez les enfants [13, 17]. Dans une région de Touba (Sénégal), Sy O *et al.* ont rapporté que les très fortes densités parasitaires (> 50000 trophozoites / μl de sang) ont été relativement plus élevés chez les enfants de moins de 14 ans. Mais la région de Touba est plus humide que la ville de Kaédi [18]. Contrairement à la même saison, les fréquences de températures n'ont pas variées selon les saisons. L'absence de pluviométrie durant la saison pluvieuse explique en partie la similarité des variations de températures entre les deux saisons. Il existe des fièvres non palustres. Ce qui laisse présager l'existence d'étiologies des fièvres non palustres. La prévalence parasitaire n'est pas significative selon que la température corporelle était entre 38 et 39 ou > 39 (p > 0,05). Par contre, la différence est plus notable selon que le patient est fébrile ou apyrétique. Notre étude s'est déroulée dans une période où la transmission est quasi absente, cette situation ne permet pas d'apprécier l'association température corporelle et prévalence parasitaire. Les études réalisées en Mauritanie ne font pas état d'une corrélation entre la température corporelle et la prévalence et/ou la charge parasitaire. Une étude effectuée à Touba (Sénégal) en 2006 montre que le taux de prélèvements positifs a été significativement plus important chez les fébriles avec 49,6% que chez les apyrétiques avec 31,9% (p < 0,05), mais le contexte de Touba est diffèrent de Kaédi [18].

En saison pluvieuse seuls les quartiers de Modèrne et Tantadji ont été touchés. Ce dernier, quartier riverain du fleuve et également à proximité de cours d'eau, était le plus touché des onze quartiers de la ville de Kaédi. En absence de pluviométrie, la présence du fleuve ou des cours d'eau peut rendre pérenne la transmission du paludisme avec une présence continue du vecteur. Globalement dans la ville de Kaédi (zone sahélienne), en raison d'une urbanisation croissante et d'une couverture médicale relativement bonne mais couplée à une importante automédication, la transmission ne peut être que faible. L'indice plasmodique a présenté une différence entre les quartiers de résidence, ceci à l'image de certaines villes Africaines [19-21]. Il a été plus élevé dans les quartiers périphériques de Kébe et Tantadji que dans le quartier central de Wandama-Kilinkare. Cette différence entre quartier d'une même ville est classiquement expliquée par des infrastructures urbaines plus développées en centre-ville avec un niveau de vie plus élevé. La faune larvaire était plus abondante lors de la saison sèche (99%) que pendant la saison pluvieuse (1%). Ce qui est conforme à l'absence de pluviométrie au moment de l'enquête saison humide. Cette faune larvaire était globalement dominée par le genre Culex (99.6%), le taux était de 0.4% pour la faune anophélienne qui n'était rencontrée qu'en saison sèche. Ces résultats, comme ceux de la parasitologie, montrent une transmission quasi inexistante en saison pluvieuse où la pluie était absente. Pour les adultes on note deux (2) *Anopheles gambiae* en saison pluvieuse contre 16 Culex tandis qu'en saison sèche six (6) *Anopheles gambiae* contre 87 Culex. Durant cette étude nous avons trouvé une prédominance des *Anopheles* femelles. Au total, huit (8) moustiques du genre *Anopheles* ont été récoltés au cours de l'étude, ce nombre est certes faible, mais est suffisant pour permettre une transmission s'ils sont infectés. Des études nationales récentes ont montré une prédominance de l'*Anopheles gambiae* dans les trois strates épidémiologiques pendant la saison sèche froide 2011. Mais au cours de la saison pluvieuse 2013, *Anopheles gambiae, Anopheles arabiensis, Anopheles pharoensis* et *Anopheles rufipes* étaient abondants dans des zones différentes [10]. Dia *et al.* lors d'une épidémie de fièvre de la vallée du Rift, ont montré qu'*An. gambiae s.l* était l'espèce prédominante (92%) [9].

## Conclusion

Notre étude a montré l'existence du *Plasmodium falciparum* et du vecteur Anopheles femelle dans la ville de Kaédi malgré l'absence de pluviométrie. Par ailleurs, il ressort de notre étude une transmission faible du paludisme au niveau de Kaédi, ceci confirme les résultats déjà décrits au niveau de la vallée du fleuve Sénégal. Ainsi les autorités sanitaires, avec des mesures de luttes efficaces et une politique sanitaire rigoureuse, pourront entrevoir dans un avenir proche une préélimination du paludisme au niveau des wilayas sud du pays. Ainsi, le diagnostic du paludisme dans les hôpitaux des wilayas du pays devra se reposer principalement sur la goutte épaisse et le frottis mince. Le test de diagnostic rapide doit s'élargir aux postes de santé et au niveau des agents de santé communautaires. Donc la confirmation biologique devra être systématique. Le contrôle de toutes les lames positives et de 10% des lames négatives doit être effectué au niveau du Laboratoire de Parasitologie-Mycologie de l'Institut National de Recherches en Santé Publique (INRSP).

### Etat des connaissances actuelles sur le sujet

- L'indice plasmodique chez la population fébrile;
- Les espèces plasmodiales circulantes;
- La composition de la population culicidienne.

### Contribution de notre étude à la connaissance

- La prévalence (indice plasmodique) du paludisme au niveau des ménages et la démonstration de la faiblesse de celle-ci favorise son éradication;
- L'ampleur des vecteurs et la corrélation de l'indice plasmodique dans la transmission à Kaédi;
- La corrélation entre la température corporelle et positivité des gouttes épaisses à Kaédi par conséquent en Mauritanie.

## Conflits d’intérêts

Les auteurs ne déclarent aucun conflit d'intérêts.
